# Supplementary material for: Epidemiological Survey of Four Reproductive Disorder Associated Viruses of Sows in Hunan Province during 2019–2021
Source: Vet Sci. 2022 Aug 11;9(8):425. doi: 10.3390/vetsci9080425 (PMC9416293; doi:10.3390/vetsci9080425)
Supplement: Supplementary file 1 [file vetsci-09-00425-s001.zip › Supplementary Table S1.pdf]

Supplementary Table S1. Detail information of PRRSV strains obtained in the present study and reference strains, including strain name, isolated country and year, GenBank accession number, and genotype, etc.,

Strain name	Country	Year	Accession No.	Length nt/aa	Genotype
Lelystad	Netherlands		M96262	606/201	PRRSV1
QYYZ	China	2011	JQ308798	603/200	PRRSV2-Lineage 3
GM2	China	2011	JN662424	603/200	PRRSV2-Lineage 3
NADC30	China	2017	MH500776	600/199	PRRSV2-Lineage 1
HLJ	China	2017	MH422084	603/200	PRRSV2-Lineage 1
HLJ	China	2019	OL546279	603/200	PRRSV2-Lineage 1
HeN	China	2016	MT036940	603/200	PRRSV2-Lineage 1
VR2332	USA	-	EF536003	603/200	PRRSV2-Lineage 5
FJTK	China	2017	KY798942	603/200	PRRSV2-Lineage 5
SDWH	China	2019	MN642104	603/200	PRRSV2-Lineage 5
JBUN	China	2019	MW717134	603/200	PRRSV2-Lineage 5
JXA1	China	2006	EF112445	603/200	PRRSV2-Lineage 8
HuN	China	2007	EF517962	603/200	PRRSV2-Lineage 8
TJ	China	2008	EU860248	603/200	PRRSV2-Lineage 8
Gansu	China	2017	MN064235	603/200	PRRSV2-Lineage 8
SC	China	2017	KY620028	603/200	PRRSV2-Lineage 8
15ZJ2	China	2016	KX815433	603/200	PRRSV2-Lineage 8
HuN-CS-2019-A	China	2019	ON968569	603/200	PRRSV2-Lineage 8
HuN-CS-2019-B	China	2019	ON968570	603/200	PRRSV2-Lineage 8
HuN-HH-2019	China	2019	ON968571	603/200	PRRSV2-Lineage 8
HuN-ZZ-2019	China	2019	ON968560	603/200	PRRSV2-Lineage 1
HuN-YY-2019-A	China	2019	ON968572	603/200	PRRSV2-Lineage 8
HuN-YY-2019-B	China	2019	ON968593	603/200	PRRSV2-Lineage 5
HuN-HY-2019	China	2019	ON968573	603/200	PRRSV2-Lineage 8
HuN-SY-2019	China	2019	ON968594	603/200	PRRSV2-Lineage 5
HuN-CD-2019	China	2019	ON968561	603/200	PRRSV2-Lineage 1
HuN-ZJJ-2019	China	2019	ON968574	603/200	PRRSV2-Lineage 8
HuN-CS-2020-A	China	2020	ON968562	603/200	PRRSV2-Lineage 1
HuN-CS-2020-B	China	2020	ON968575	603/200	PRRSV2-Lineage 8
HuN-HY-2020	China	2020	ON968576	603/200	PRRSV2-Lineage 8
HuN-ZZ-2020	China	2020	ON968563	603/200	PRRSV2-Lineage 1
HuN-XT-2020	China	2020	ON968577	603/200	PRRSV2-Lineage 8
HuN-SY-2020	China	2020	ON968578	603/200	PRRSV2-Lineage 8
HuN-YY-2020	China	2020	ON968579	603/200	PRRSV2-Lineage 8
HuN-CD-2020-A	China	2020	ON968564	603/200	PRRSV2-Lineage 1
HuN-CD-2020-B	China	2020	ON968580	603/200	PRRSV2-Lineage 8
HuN-ZJJ-2020	China	2020	ON968581	603/200	PRRSV2-Lineage 8
HuN-XX-2020	China	2020	ON968582	603/200	PRRSV2-Lineage 8
HuN-LD-2020	China	2020	ON968565	603/200	PRRSV2-Lineage 1
HuN-YiY-2020-A	China	2020	ON968583	603/200	PRRSV2-Lineage 8
HuN-YiY-2020-B	China	2020	ON968584	603/200	PRRSV2-Lineage 8
HuN-CS-2021	China	2021	ON968595	603/200	PRRSV2-Lineage 5

HuN-HY-2021-A	China	2021	ON968585	603/200	PRRSV2-Lineage 8
HuN-HY-2021-B	China	2021	ON968596	603/200	PRRSV2-Lineage 5
HuN-ZZ-2021	China	2021	ON968586	603/200	PRRSV2-Lineage 8
HuN-XT-2021-A	China	2021	ON968587	603/200	PRRSV2-Lineage 8
HuN-XT-2021-B	China	2021	ON968566	603/200	PRRSV2-Lineage 1
HuN-SY-2021	China	2021	ON968588	603/200	PRRSV2-Lineage 8
HuN-YY-2021	China	2021	ON968589	603/200	PRRSV2-Lineage 8
HuN-CD-2021	China	2021	ON968567	603/200	PRRSV2-Lineage 1
HuN-ZJJ-2021-A	China	2021	ON968590	603/200	PRRSV2-Lineage 8
HuN-ZJJ-2021-B	China	2021	ON968568	603/200	PRRSV2-Lineage 1
HuN-XX-2021	China	2021	ON968591	603/200	PRRSV2-Lineage 8
HuN-LD-2021	China	2021	ON968592	603/200	PRRSV2-Lineage 8
