# Supplementary material for: Epidemiological Survey of Four Reproductive Disorder Associated Viruses of Sows in Hunan Province during 2019–2021
Source: Vet Sci. 2022 Aug 11;9(8):425. doi: 10.3390/vetsci9080425 (PMC9416293; doi:10.3390/vetsci9080425)
Supplement: Supplementary file 1 [file vetsci-09-00425-s001.zip › Supplementary Table S2.pdf]

| Strain name | Country | Year | Accession No. | Length nt/aa | Genotype |
|---|---|---|---|---|---|
| SH1801 | China | 2018 | OM032622 | 705/234 | PCV2d |
| XJ01 | China | 2016 | KX845692 | 702/233 | PCV2b |
| LX | China | 2016 | KX845693 | 705/234 | PCV2d |
| SZ | China | 2016 | KX845694 | 702/233 | PCV2b |
| HBBD160823 | China | 2016 | KY126312 | 705/234 | PCV2d |
| CHHBCZ | China | 2018 | MG786933 | 702/233 | PCV2e |
| GX0601 | China | 2009 | EF524532 | 702/233 | PCV2e |
| CHHBDZ | China | 2018 | MG786934 | 702/233 | PCV2b |
| CHHBRC | China | 2018 | MG798696 | 702/233 | PCV2a |
| BF | China | 2005 | GQ404853 | 702/233 | PCV2a |
| JF | China | 2010 | HM038022 | 702/233 | PCV2b |
| JZ | China | 2005 | DQ206444 | 702/233 | PCV2b |
| MN614 | USA | 2010 | GQ404852 | 702/233 | PCV2b |
| DK1980PMWS | Denmark | 2008 | EU148503 | 705/234 | PCV2c |
| DK1987PMWS | Denmark | 2008 | EU148504 | 705/234 | PCV2c |
| Buffalo2 | China | 2015 | KM116514 | 705/234 | PCV2d |
| GXWM | China | 2007 | EF675241 | 705/234 | PCV2d |
| UFV1 | Brzail | 2014 | KJ187306 | 705/234 | PCV2d |
| GD18 | China | 1999 | MF278779 | 705/234 | PCV2f |
| YN-8 | China | 2011 | HM776452 | 705/234 | PCV2f |
| HuN-CS-2019 | China | 2019 | ON968536 | 702/233 | PCV2d |
| HuN-HH-2019 | China | 2019 | ON968557 | 702/233 | PCV2b |
| HuN-ZZ-2019 | China | 2019 | ON968537 | 702/233 | PCV2d |
| HuN-YY-2019 | China | 2019 | ON968538 | 705/234 | PCV2d |
| HuN-HY-2019 | China | 2019 | ON968539 | 705/234 | PCV2d |
| HuN-CS-2020 | China | 2020 | ON968540 | 705/234 | PCV2d |
| HuN-HY-2020 | China | 2020 | ON968558 | 702/233 | PCV2b |
| HuN-ZZ-2020 | China | 2020 | ON968541 | 705/234 | PCV2d |
| HuN-XT-2020 | China | 2020 | ON968542 | 705/234 | PCV2d |
| HuN-SY-2020 | China | 2020 | ON968559 | 702/233 | PCV2b |
| HuN-YY-2020 | China | 2020 | ON968543 | 705/234 | PCV2d |
| HuN-ZJJ-2020 | China | 2020 | ON968544 | 705/234 | PCV2d |
| HuN-CS-2021-A | China | 2021 | ON968545 | 705/234 | PCV2d |
| HuN-CS-2021-B | China | 2021 | ON968546 | 705/234 | PCV2d |
| HuN-ZZ-2021 | China | 2021 | ON968547 | 705/234 | PCV2d |
| HuN-HY-2021 | China | 2021 | ON968548 | 702/233 | PCV2d |
| HuN-XT-2021 | China | 2021 | ON968549 | 705/234 | PCV2d |
| HuN-SY-2021 | China | 2021 | ON968550 | 705/234 | PCV2d |
| HuN-YY-2021 | China | 2021 | ON968551 | 702/233 | PCV2d |
| HuN-CD-2021 | China | 2021 | ON968552 | 705/234 | PCV2d |
| HuN-ZJJ-2021-A | China | 2021 | ON968553 | 705/234 | PCV2d |
| HuN-ZJJ-2021-B | China | 2021 | ON968554 | 705/234 | PCV2d |
| HuN-XX-2021 | China | 2021 | ON968555 | 702/233 | PCV2d |

| | | | | | |
|---|---|---|---|---|---|
| HuN-LD-2021 | China | 2021 | ON968556 | 702/233 | PCV2d |

**Supplementary Table S2.** Detail information of PCV2 strains obtained in the present study and reference strains, including strain name, isolated country and year, GenBank accession number, and genotype, etc.,
