# Supplementary material for: Epidemiological Survey of Four Reproductive Disorder Associated Viruses of Sows in Hunan Province during 2019–2021
Source: Vet Sci. 2022 Aug 11;9(8):425. doi: 10.3390/vetsci9080425 (PMC9416293; doi:10.3390/vetsci9080425)
Supplement: Supplementary file 1 [file vetsci-09-00425-s001.zip › Supplementary Table S3.pdf]

Supplementary Table S3. Detail information of CSFV strains obtained in the present study and reference strains, including strain name, isolated country and year, GenBank accession number, and genotype, etc.,

Strain name	Country	Year	Accession No.	Length nt/aa	Genotype
Shimen	China	2008	FJ598612	1119/373	1.1
HCLV	Africa	2002	AF091507	1119/373	1.1
RUCSFPLUM	Africa	2004	AY578688	1119/373	1.2
CSF0306	Malaysia	1986	JQ411570	1119/373	1.3
CSF0705	Cuba	1958	JX028201	1119/373	1.4
CSF1057	Cuba	2011	JX028203	1119/373	1.4
96TD	China	2004	AY554397	1119/373	2.1a
Italy E2	Italy	2001	AY027672	1119/373	2.1a
HuB	China	2011	KC809979	1119/373	2.1b
HLJZZ	China	2014	KU375260	1119/373	2.1b
JLHD	China	2012	KU375252	1119/373	2.1b
HNSY	China	2012	JX898524	1119/373	2.1c
HY92E2	Vietnam	2016	MF977829	1119/373	2.1c
GD53	China	2011	KP343640	1119/373	2.1c
HNLY	China	2011	JX262391	1119/373	2.1c
HNCS	China	2011	JX898523	1119/373	2.1c
SDTA4	China	2015	KT953610	1119/373	2.1d
SDXT	China	2015	KT953611	1119/373	2.1d
LN184E2	China	2006	DQ907717	1119/373	2.2
LAL-290E2	India	2012	KC851953	1119/373	2.2
Roesrach	Germany	2009	GU233734	1119/373	2.3
ND21E2	Korea	2014	KP702210	1119/373	2.3
HuN-CS-2019	China	2019	ON968597	1119/373	2.1c
HuN-ZZ-2019	China	2019	ON968600	1119/373	2.1b
HuN-ZZJ-2019	China	2019	ON968601	1119/373	2.1b
HuN-YY-2020	China	2020	ON968602	1119/373	2.1b
HuN-ZJJ-2020	China	2020	ON968603	1119/373	2.1b
HuN-CS-2020	China	2020	ON968604	1119/373	2.1b
HuN-HY-2020	China	2020	ON968598	1119/373	2.1c
HuN-ZZ-2021	China	2021	ON968605	1119/373	2.1b
HuN-XT-2021	China	2021	ON968606	1119/373	2.1b
HuN-SY-2021	China	2021	ON968607	1119/373	2.1b
HuN-YY-2021	China	2021	ON968599	1119/373	2.1c
HuN-ZJJ-2021	China	2021	ON968608	1119/373	2.1b
HuN-CS-2021	China	2021	ON968609	1119/373	2.1b
