# Supplementary material for: Epidemiological Survey of Four Reproductive Disorder Associated Viruses of Sows in Hunan Province during 2019–2021
Source: Vet Sci. 2022 Aug 11;9(8):425. doi: 10.3390/vetsci9080425 (PMC9416293; doi:10.3390/vetsci9080425)
Supplement: Supplementary file 1 [file vetsci-09-00425-s001.zip › Supplementary Table S4.pdf]

Supplementary Table S4. Detail information of PRV strains obtained in the present study and reference strains, including strain name, isolated country and year, GenBank accession number, and genotype, etc.,

Strain name	Country	Year	Accession No.	Length nt/aa	Genotype
SC	China	1986	KT809429	1737/578	Genotype II
Ea	China	1993	KX423960	1737/578	Genotype II
Fa	China	2001	KM189913	1737/578	Genotype II
TJ	China	2012	KJ789182	1740/579	Genotype II
Kaplan	Hungary	-	JF797218	1734/577	Genotype I
Becker	USA	-	JF797219	1734/577	Genotype I
Kolchis	Greece	2010	KT983811	1734/577	Genotype I
HeN1	China	2012	KP098534	1740/579	Genotype II
BJ-YT	China	2012	KC981239	1740/579	Genotype II
JS-2012	China	2012	KP722022	1740/579	Genotype II
hSD-1	China	2019	MT468550	1740/579	Genotype II
HuN-XX	China	2020	MZ501784	1740/579	Genotype II
HuN-LD	China	2019	MZ501783	1740/579	Genotype II
HuN-XT	China	2020	MZ501782	1740/579	Genotype II
HuN-YY	China	2018	MZ501781	1737/578	Genotype II
HuN-CS-2019	China	2019	ON968530	1740/579	Genotype II
HuN-ZJJ-2020	China	2020	ON968531	1740/579	Genotype II
HuN-HH-2020	China	2019	ON968532	1740/579	Genotype II
HuN-ZZ-2020	China	2019	ON968533	1740/579	Genotype II
HuN-YY-2021	China	2019	ON968534	1740/579	Genotype II
HuN-LD-2021	China	2019	ON968535	1740/579	Genotype II
